# Characterization of Thick Selenium Layers for Dual-Layer X-ray Imaging

**DOI:** 10.1109/tns.2025.3650559

**Published:** 2026-01-05

**Authors:** Akyl Swaby, Kaitlin Hellier, Linxi Shi, Adam S. Wang, Shiva Abbaszadeh

**Affiliations:** Department of Electrical and Computer Engineering, University of California Santa Cruz, Santa Cruz, CA 95064, USA; Department of Electrical and Computer Engineering, University of California Santa Cruz, Santa Cruz, CA 95064, USA; Department of Radiology, Stanford University School of Medicine, Stanford, CA 94305, USA.; Department of Radiology, Stanford University School of Medicine, Stanford, CA 94305, USA.; Department of Electrical and Computer Engineering, University of California Santa Cruz, Santa Cruz, CA 95064, USA

**Keywords:** Amorphous Selenium, Cascade Linear System Model, Charge transport, Dual Energy, Temporal characteristics, X-ray imaging

## Abstract

Amorphous selenium (a-Se) as a direct conversion X-ray photoconductor has many desirable properties in X-ray imaging, due to its high spatial resolution, low dark current, high absorption efficiency, and large area fabrication. We present the fabrication and performance evaluation of thick a-Se layers for a cost-effective dual-layer X-ray flat-panel detector (DL-FPD). Building on a Cascaded Linear Systems Model that identifies 200–400 μm as the optimal a-Se thickness for the top layer detector, we manufactured 253 μm and 414 μm a-Se samples with a polyimide hole-blocking contact to suppress dark current to below 10 pA/mm^2^ at electric fields up to 10 V/μm. Under low-energy X-ray irradiation, both devices demonstrated increasing response to increasing tube energy, with the 414 μm layer achieving higher photocurrent due to increased photon absorption. Temporal lag analysis revealed more pronounced signal persistence in the thicker sample at a lower bias, consistent with increased carrier trapping for greater thicknesses. These measurements provide critical metrics such as leakage, photoresponse, and lag for guiding the design of readout electronics in future direct/indirect DL-FPD arrays.

## I. Introduction

Amorphous selenium (a-Se) (Z=34) is one of the most widely used X-ray photoconductor materials in direct conversion flat-panel detectors (FPD) for low energy imaging due to its desirable properties such as high x-ray absorption, high spatial resolution, high conversion efficiencies, low dark current and electronic noise, and compatibility with large-area thin film transistor (TFT) fabrication [1]. The most common applications for which it has been commercialized fall within medical imaging, such as general radiography, fluoroscopy, and mammography, though it finds uses in additional fields such as high-energy physics, memory and switching devices, bio and industrial imaging. To extend FPD capabilities, dual-layer (DL) and multi-layer architectures have been proposed, enabling single-exposure spectral separation with high resolution, motion-artifact suppression, and dose efficiency [2]–[7]. In DL FPDs, low-energy (LE) and high-energy (HE) images are acquired simultaneously, facilitating dual-energy (DE) material discrimination of soft tissue, bone, contrast agents, and calcification in a single polyenergetic exposure [8]–[12]. Previously we proposed a direct/indirect conversion DL FPD design combining a-Se top layer with CsI:Tl bottom layer to enhance DE performance, spatial resolution, and dose efficiency relative to existing DE detectors [13].

A cascaded linear systems model (CLSM) was previously developed to quantify signal and noise propagation in detector architectures and to optimize layer design for specific tasks, such as coronary artery calcium (CAC) detection using weighted log-subtraction DE decomposition [14], [15]. That analysis identified an a-Se top-layer thickness of 200 – 400 μm as the optimal trade-off among dose efficiency, spatial resolution, and DE sensitivity. Thick a-Se layers (*≈* 200 μm) have been specifically investigated for digital mammography to improve X-ray absorption efficiency at low photon energies, typically below 35 keV [16]–[18]. By directly converting incident X-ray photons into electrical charges without the intermediate step of light emission, a-Se detectors offer high spatial resolution (>0.8 at 6 cycles/mm for an 85 μm pixel pitch), which is critical for detecting microcalcifications and other subtle features in breast tissue [19], [20]. Its high X-ray absorption efficiency at mammographic energies allows for high detective quantum efficiency (>0.6 at 2 cycles/mm, >0.2 at 6 cycles/mm) and enables lower patient radiation doses while preserving diagnostic image quality [18]. The ability to deposit uniform a-Se layers over large areas facilitates the production of full-field digital mammography detectors with small pixel sizes and high reliability, further enhancing diagnostic accuracy and workflow efficiency in breast imaging applications [21].

Early implementations of thick a-Se layers in mammography and general radiography demonstrated that uniform charge transport and low dark current levels could be achieved through precise deposition optimization and advanced blocking-contact engineering [22], [23]. In this work, we fabricated 200–400 μm a-Se layers and evaluate their fundamental performance to guide the development of a DL imager. This study demonstrates our capability to produce thick selenium layers and provide critical data to inform readout electronic design, providing a foundation for the future development of a practical DL imager.

## II. Methods

### A. Fabrication

The thermal evaporator in the Radiological Instrumentation Laboratory (RIL) is capable of depositing a maximum of 250 μm thick layer of a-Se uniformly over a 4.5 inch diameter when the crucible has been filled [24], [25]. Single-pixel devices of stabilized a-Se (0.2% As, 10 ppm Cl) were fabricated on clean indium tin oxide (ITO) substrates, with and without a polyimide blocking layer [26], with thicknesses ranging from 199 to 414 μm. ITO-coated glass substrates were cleaned by ultrasonication for 10 minutes in acetone, then isopropyl alcohol, then rinsed with deionized water and dried with nitrogen. A 1 μm polyimide blocking layer (PI2610) was spin-coated onto the substrate at 1450 RPM for 90 seconds, dried at 90°C for 1–2 minutes, then cured by ramping at 4°C/min to 350°C and held at that temperature for 30 minutes. Devices were allowed to cool to room temperature for 1 hour. Stabilized a-Se was deposited in a dedicated Se thermal evaporator at a rate of 90 Å/s, rotation speed of 40 RPM, and substrate temperature of 65°C. The substrate was shuttered during the heating of the evaporation material. Once the target rate of 90 Å/s was achieved, 1–3 μm of material was evaporated before opening the shutter to ensure consistent material evaporation. The shutter was then opened, and deposition occurred for several hours until the evaporation rate and deposition pressure could no longer be maintained at a stable value, at which time the shutter was closed. A small amount of residual melted material remained post-deposition, ensuring that the crucible was never fully emptied.. Gold electrodes 100 nm thick were deposited by electron beam evaporation. Devices were fully encapsulated with an HV epoxy after HV and signal wires were attached to the electrodes.

### B. Characterization

**Fig. 1.**
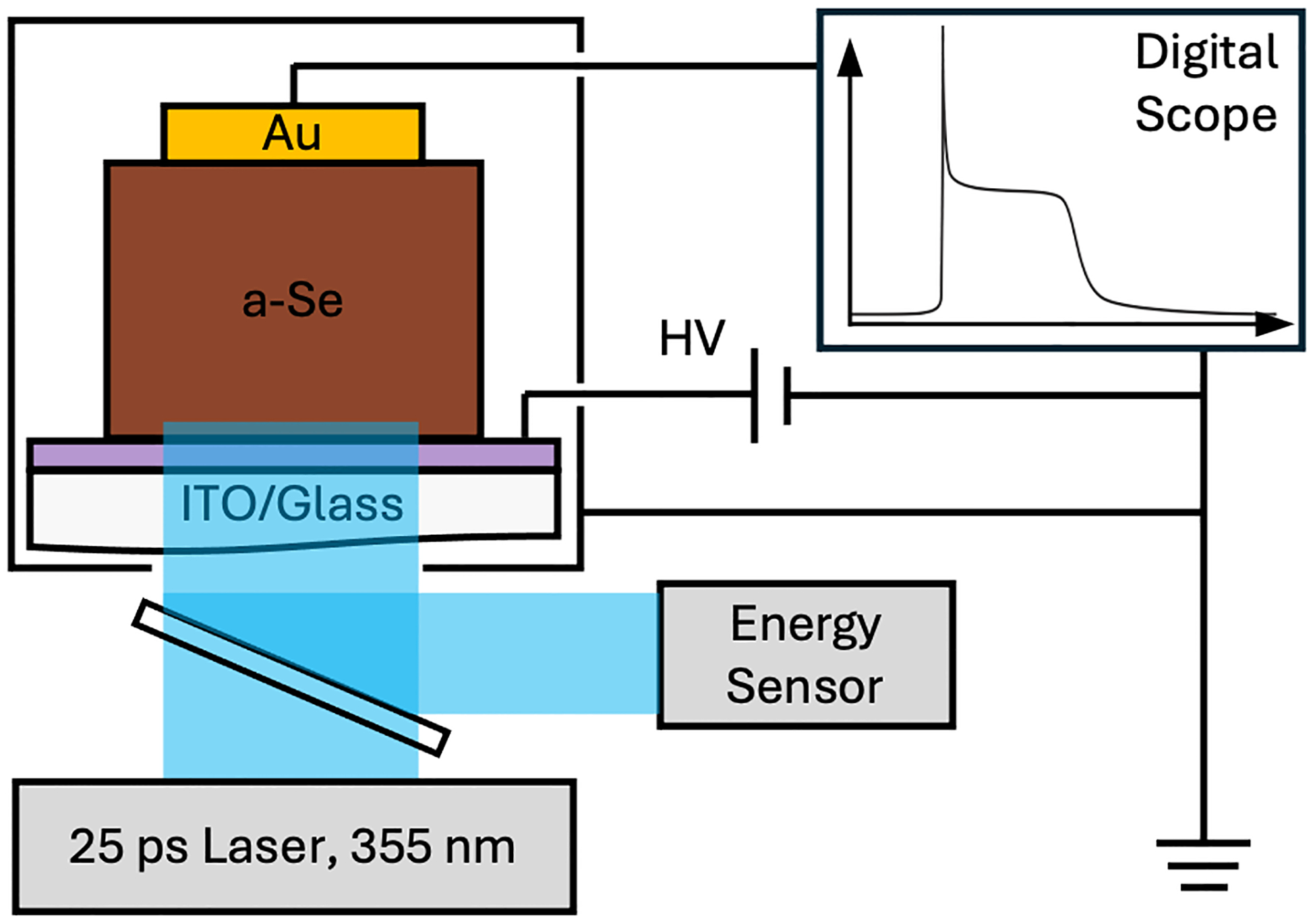

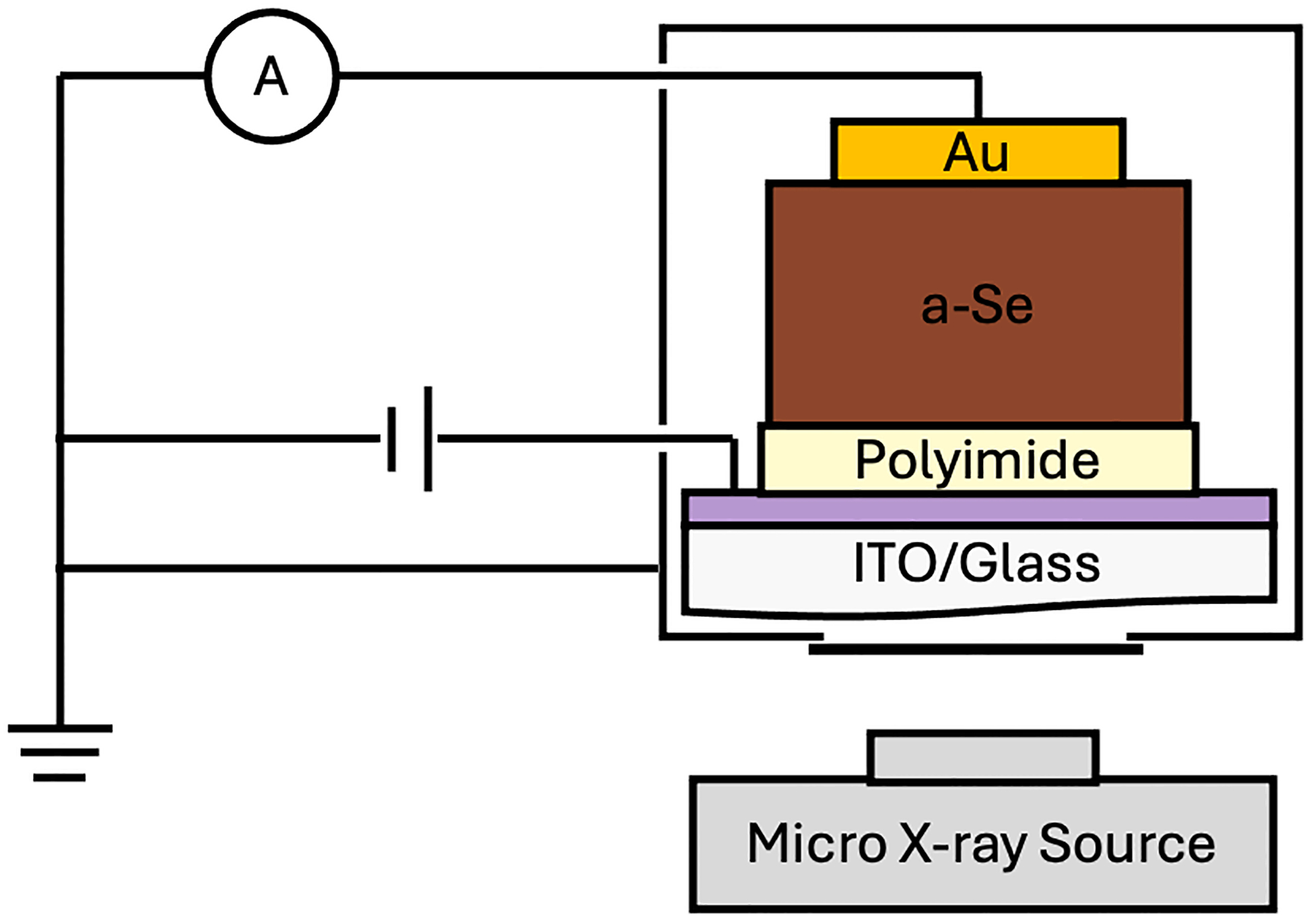
Experimental setups for (a) time of flight measurements and (b) X-ray and dark current measurements.

Devices were characterized by transient photocurrent time of flight (TOF) to determine the effective hole carrier drift mobility, μ, following the equation

(1) $$\mu =\frac{L^{2}}{V\cdot t_{T}},$$

where *L* is the thickness, *V* the applied voltage, and *t*_*t*_ the carrier transit time determined from the measurement [27]. A 25 ps, 355 nm pulsed laser (Ekspla) was used to excite the sample. The beam passed through a beam splitter to measure the intensity of the pulse, then was attenuated using neutral density filters to ensure small signal conditions were met. The signal was read out through a digital oscilloscope (Keysight DSOS404A). A schematic of the experimental setup can be seen in Figure 1a. The transit time was determined as the time from the initial pulse to when the signal plateau was reduced by 50%, as described for multiple-trap transport in Kasap (2022) [28].

Current measurements were performed by applying a positive DC bias to the ITO from a high-voltage power supply (Weiner HV module) and reading out the signal from the Au electrode on a picoammeter (Keithley Model 6487). The sample was sealed in a grounded aluminum box, with a blacked-out gap in the aluminum to expose the detector through the ITO contact. Measurements were performed on 3 mm and 4 mm diameter devices. Dark current measurements were found by averaging the value before exposure, after the device had been allowed to settle for at least 10 minutes after the bias was applied. An X-ray tube (MXR MicroBox Integrated X-Ray Source) with a source to image distance (SID) of 30.5 cm with 0.25 mm Be filtration was used to evaluate photocurrent response at tube voltages of 40, 60, and 80 kV, with a total power of 8 W. Exposure was performed by setting tube parameters, turning on the source, then exposing for 4 seconds once the tube voltage was reached. Photocurrent values were calculated as an average over those 4 seconds. Devices were allowed to recover to the original dark current level before proceeding to the next tube voltage or increasing bias. A schematic of the experimental setup can be seen in Fig. 1b.

## III. Results & Discussion

To adequately absorb energies required for the LE (<35 keV) X-rays, a-Se thicknesses greater than 100 μm must be fabricated. However, evaporation crucibles have limited capacity and require reloading of the crucible material to achieve higher thicknesses, which is performed by opening the chamber and breaking the vacuum mid-fabrication. In previous work from this group [24], the effects of breaking vacuum showed no significant effects on the transport properties and established a range of effective drift mobility values observed across both literature and in our experiment. That work demonstrated that there can be variability between samples and devices and does not appear to have a strong effect on the performance of the device in photoconversion efficiency, leakage, or recovery from pulsing.

**Fig. 2.**
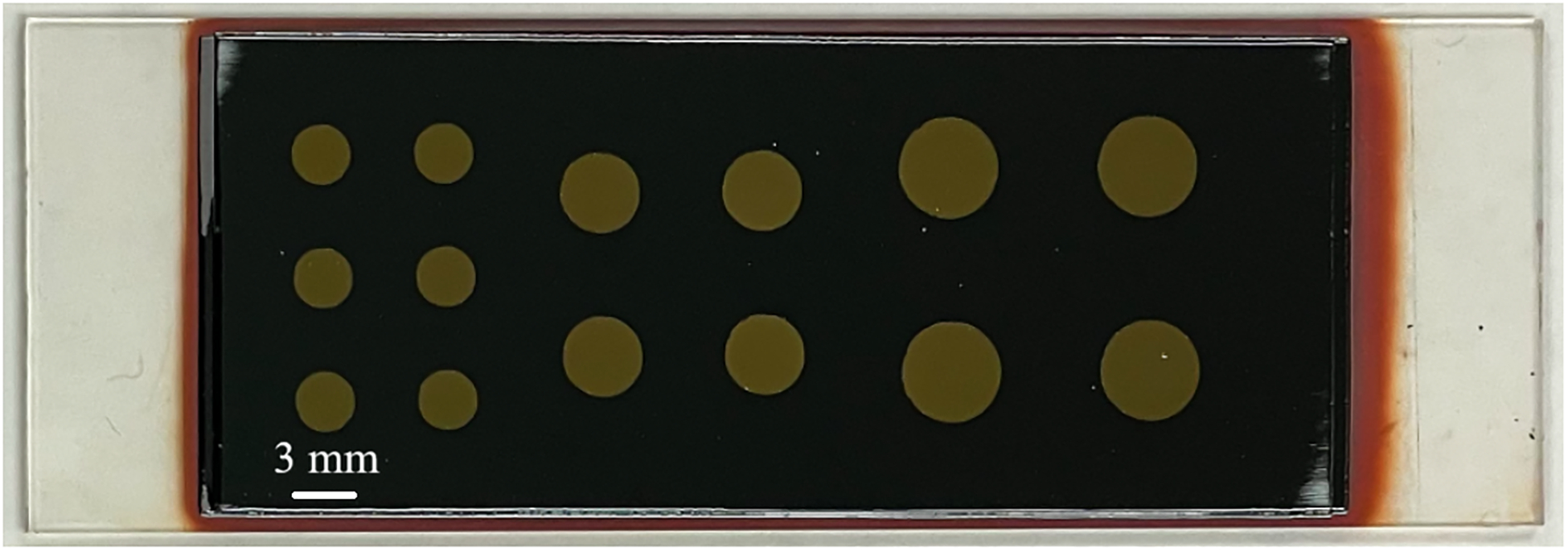
A device fabricated from two depositions of a-Se on PI, with 14 devices, prior to bonding and epoxy.

To confirm that going to even greater thicknesses will not significantly impact the performance of the device, we compared eight samples with thicknesses ranging from 100 to 414 μm. Figure 2 shows one of the fabricated multilayer devices; along the edges, two layers of a-Se can be seen.

**Fig. 3.**
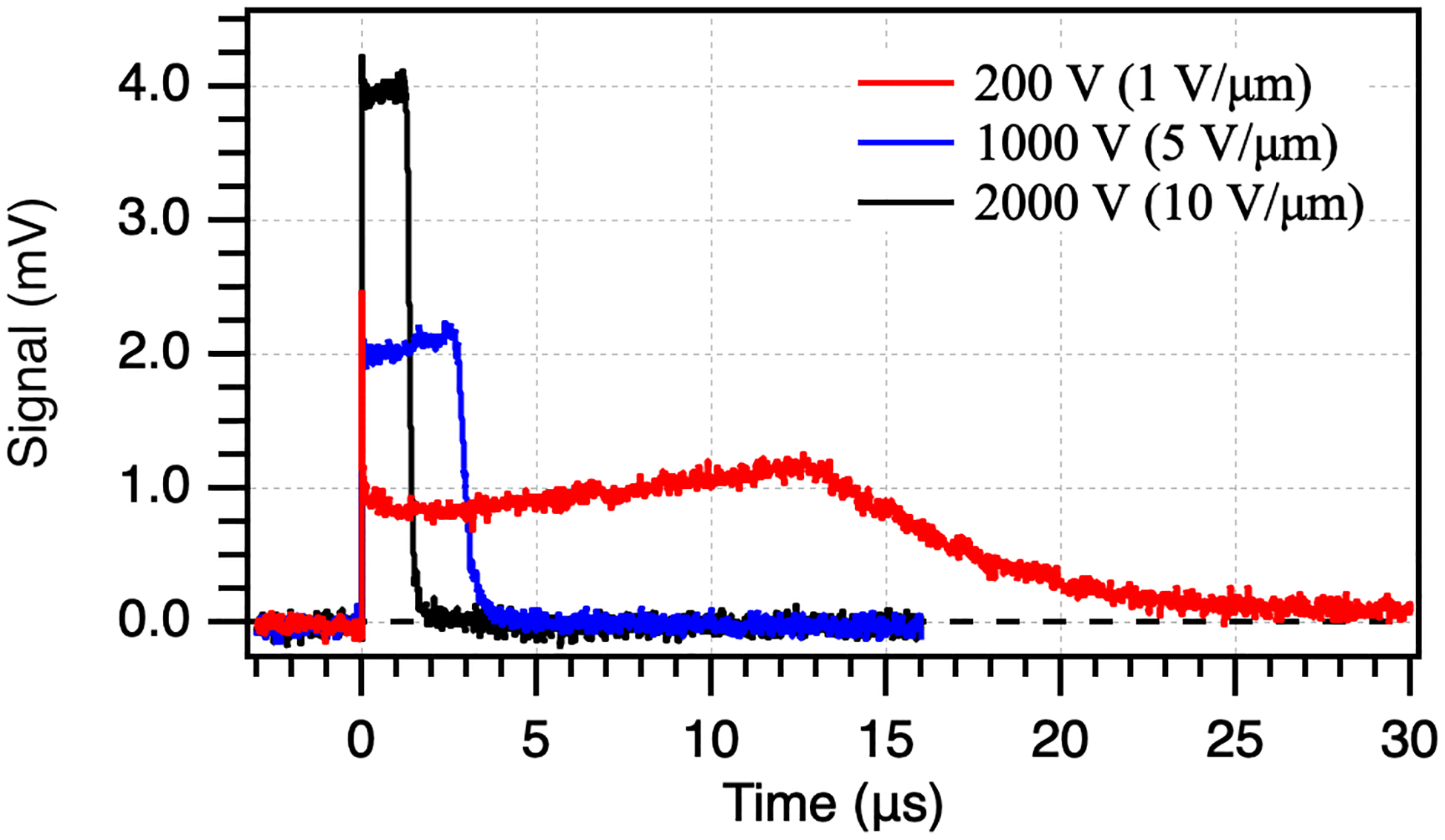

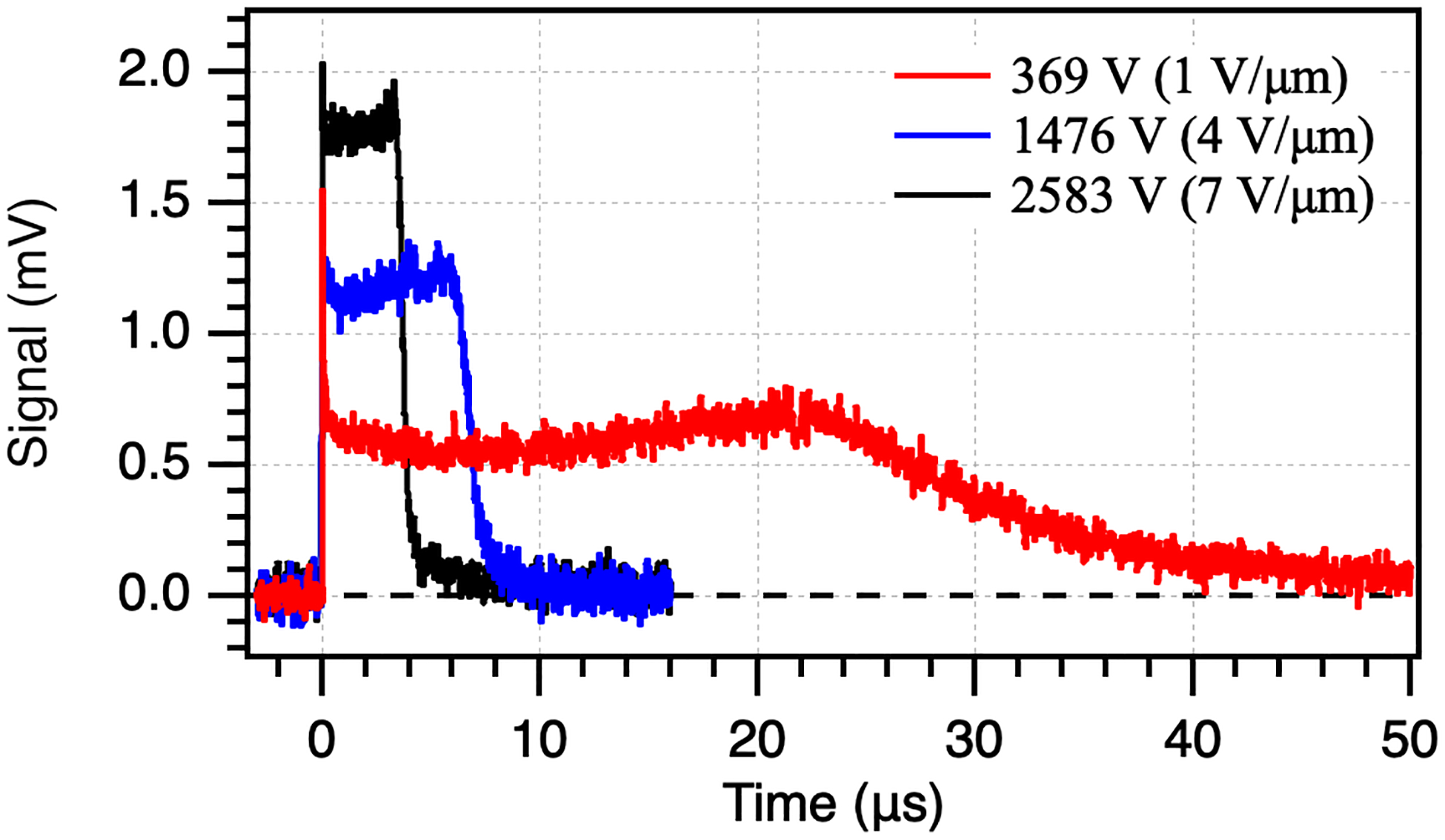
Time of flight waveforms for (a) 199 μm and (b) 369 μm thick devices at lowest, middle, and highest bias.

Transient photocurrent TOF was performed for these samples to extract effective transit times for hole carriers in the a-Se devices to ensure the effective drift mobility did not drastically decrease with increasing thickness, which may indicate a drop in sample quality with thicker layers. Fig. 3 shows the waveforms for the (a) 199 μm and (b) 369 μm thick devices at the lowest, middle, and highest fields applied. All devices at various thicknesses show similar behavior. All waveforms show a slight increase in signal before carrier collection begins, an artifact due to field distortion from carrier injection and extended time under applied bias. At 1 V/μm, a high level of dispersion can be seen in the tail of the waveforms at both thicknesses. Amorphous selenium has been shown to be dispersionless, as shallow hole traps are close to the valence band edge and participate in multiple trap transport [29]. Some dispersion may be expected due to diffusion in the material, and with increased thickness, a higher level of diffusion would occur. The high level observed may indicate issues due to space charge and field distortion from the experimental setup, or a decline in film quality with high thickness. As the field, *F*, is increased, the width of the dispersion will decrease, following an inverse proportionality as Δ*t* = Δ*x/μF*, where Δ*x* is the photocurrent spread.

**Fig. 4.**
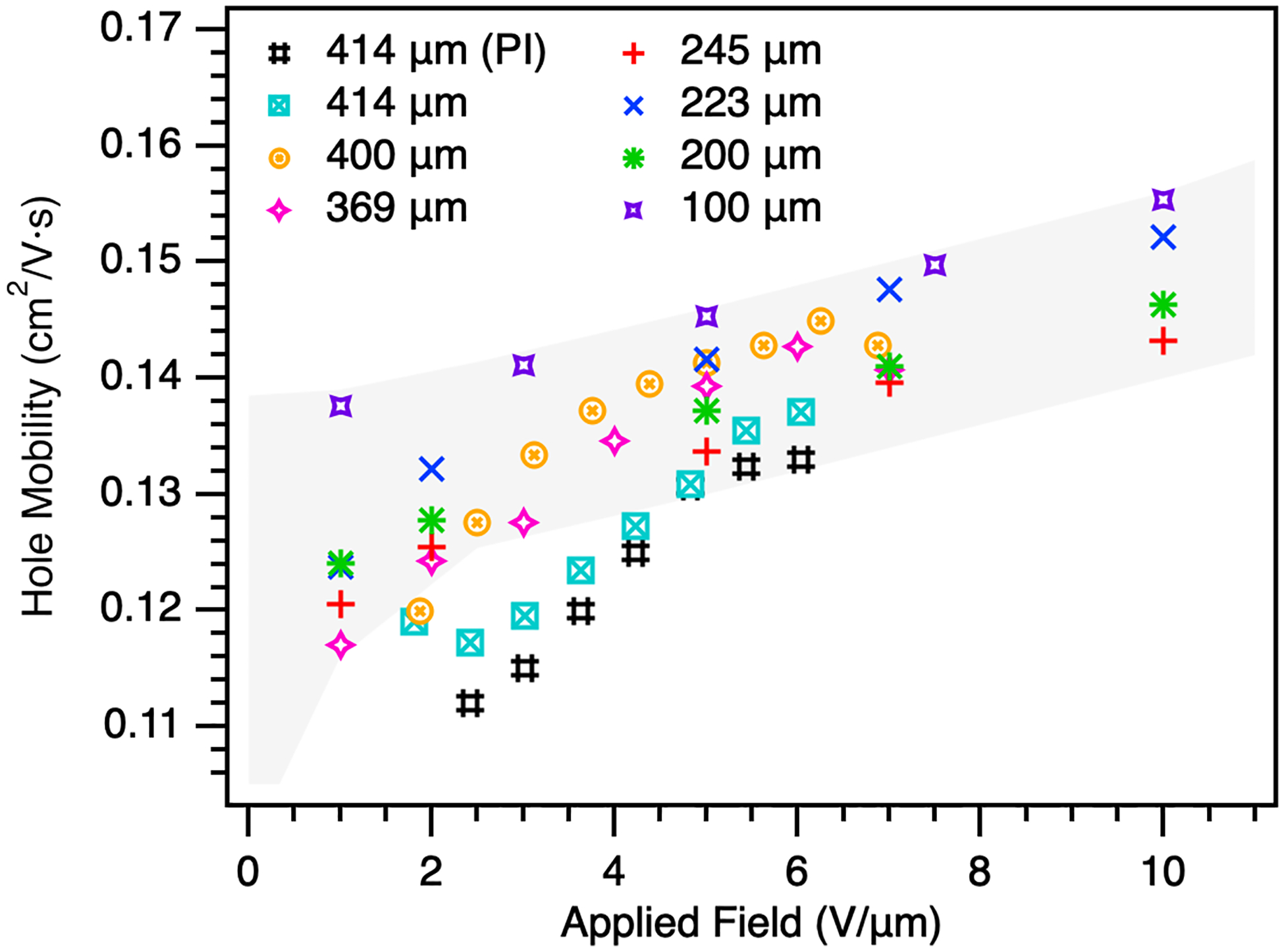
Hole mobility for several samples with thicknesses ranging from 100 to 414 μm, up to 10 V/μm. Samples were fabricated without an HBL, except for one 414 sample, which was characterized to understand the impact of the PI blocking layer.

Fig. 4 shows the calculated effective hole mobility for the samples ranging from 0 to 10 V/μm. Mobility values for samples with a-Se thicknesses ranging from 100 to 245 μm were first reported in Hellier et al. [24]. All devices show an increase in mobility with applied field, a well known feature in stabilized a-Se [30], [31]. At low fields, the calculated mobility in the thicker devices falls outside the previously established range at low fields. As the field increases to 5 V/μm and above, the mobility returns to the expected range. Though the effective mobility should be independent of thickness at a given field, it is possible the higher level of dispersion in thicker samples results in an inaccurate estimation of the observed transit time or is due to an increase in localized states, formed during the fabrication process and increases with device thickness. In the case of the latter, these may have less impact at higher fields as carriers more readily tunnel through energy barriers. To better understand this, deeper exploration of the trap mechanisms, interfaces, film quality, and effects of TOF signal distortion must be investigated, with future work utilizing interrupted-field TOF. However, for the purposes of this work, we do not see a significant reduction in the mobility, whether due to calculation or a decrease in film quality with greater thickness, and it can be recovered with the application of fields > 5 V/μm for the thickest devices. This implies that, as we target the optimal thickness required for the LE layer of the DL detector and apply fields greater than 5 V/μm, we can expect transport to be maintained.

**Fig. 5.**
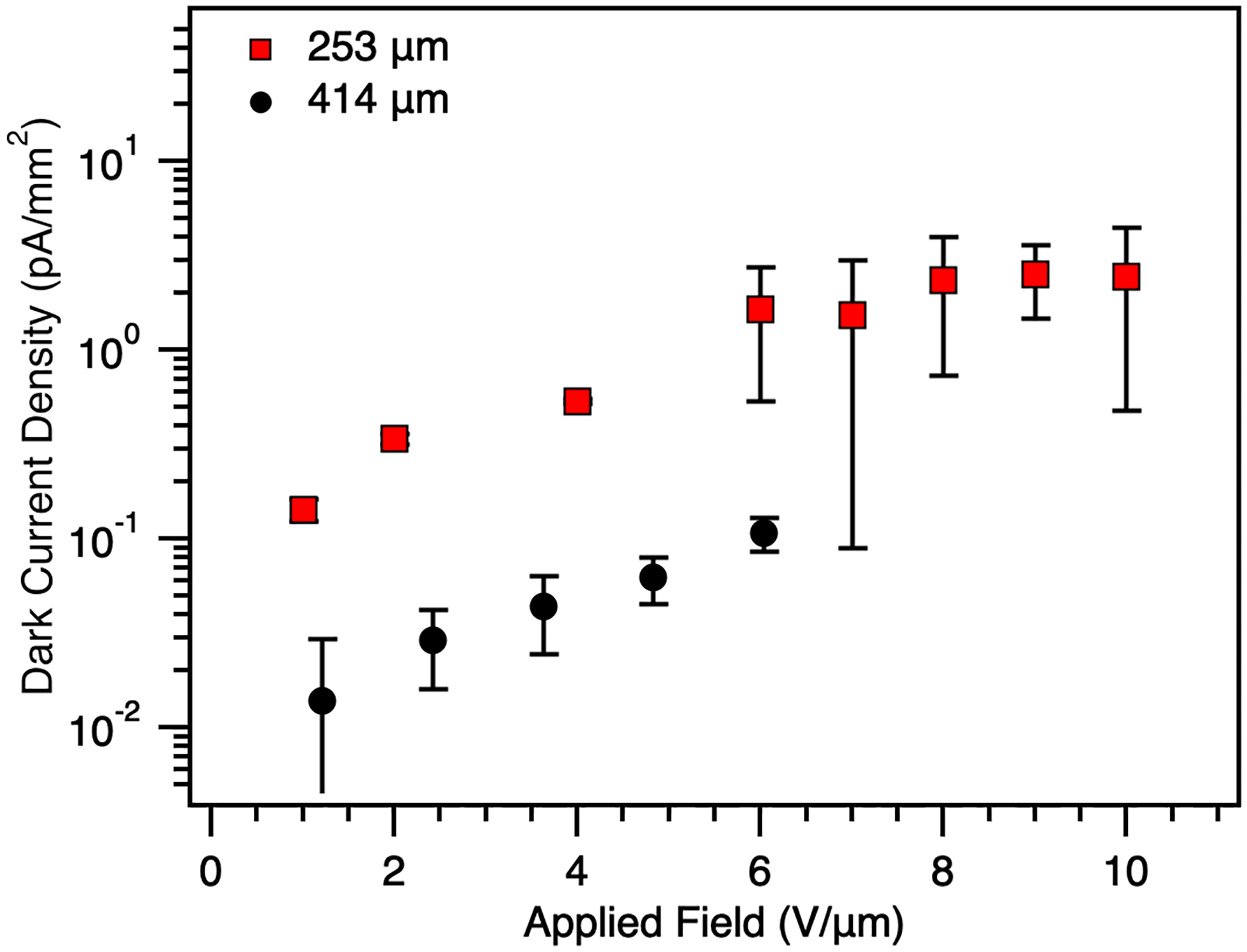
Dark current density for the 253 μm and 414 μm samples as a function of the applied field.

To obtain high-quality images and maintain a quantum noise limited system for pixelated detectors, the device dark current should be smaller than 10 pA/mm^2^ [32]. However, maintaining low dark currents at high electric fields is challenging. A high dark current reduces the dynamic range of the detector. The dominant dark current component in an a-Se detector was previously reported to be due to hole injection [33]. As a consequence, there is a need for a proper hole blocking contact that is compatible with the large area fabrication process and is able to create a good interface with a-Se. Two a-Se detectors were fabricated with a PI hole blocking layer to allow operation at high electric fields (>10 V/μm) while maintaining low dark current, without deterioration of transient performance [34]. The a-Se layers were fabricated at 253 μm and 414 μm. Fig. 5 shows a decrease in dark current as the thickness of a-Se increases. Over the range of measured applied fields (0–10 V/μm for the 253 μsample and 0–6 V/μm for the 414 μm sample), the current was maintained below 10 pA/mm^2^.

**Fig. 6.**
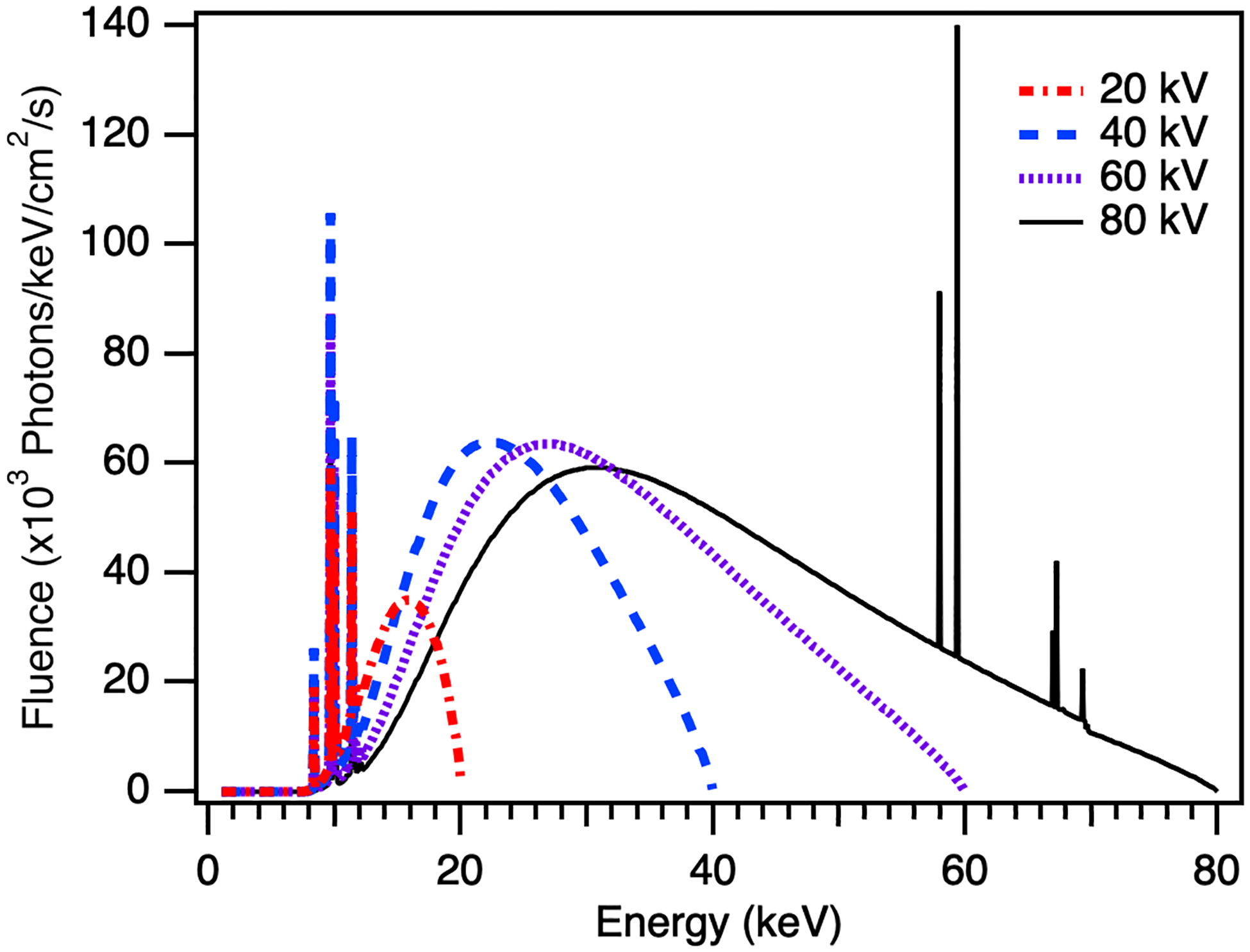
Simulated spectra for the MXR X-ray source unfiltered) at 20, 40, 60, and 80 kV tube voltages.

X-ray emission curves for 20 to 80 keV were simulated by incorporating the parameters of the X-ray source into the X-ray tube modeling program SpekPy, and are shown in Fig. 6 [35], [36]. A tube voltage of 20 kV shows a very small output; as the tube voltage increases, we see much higher fluence, and can expect higher signal. At thicknesses over 200 μm, a-Se can be expected to nearly fully absorb the spectra from a 20 kV output; we focused our studies on higher tube voltages, so that we could better understand how increasing thickness, and therefore absorption of higher energies, would impact our signal output.

**Fig. 7.**
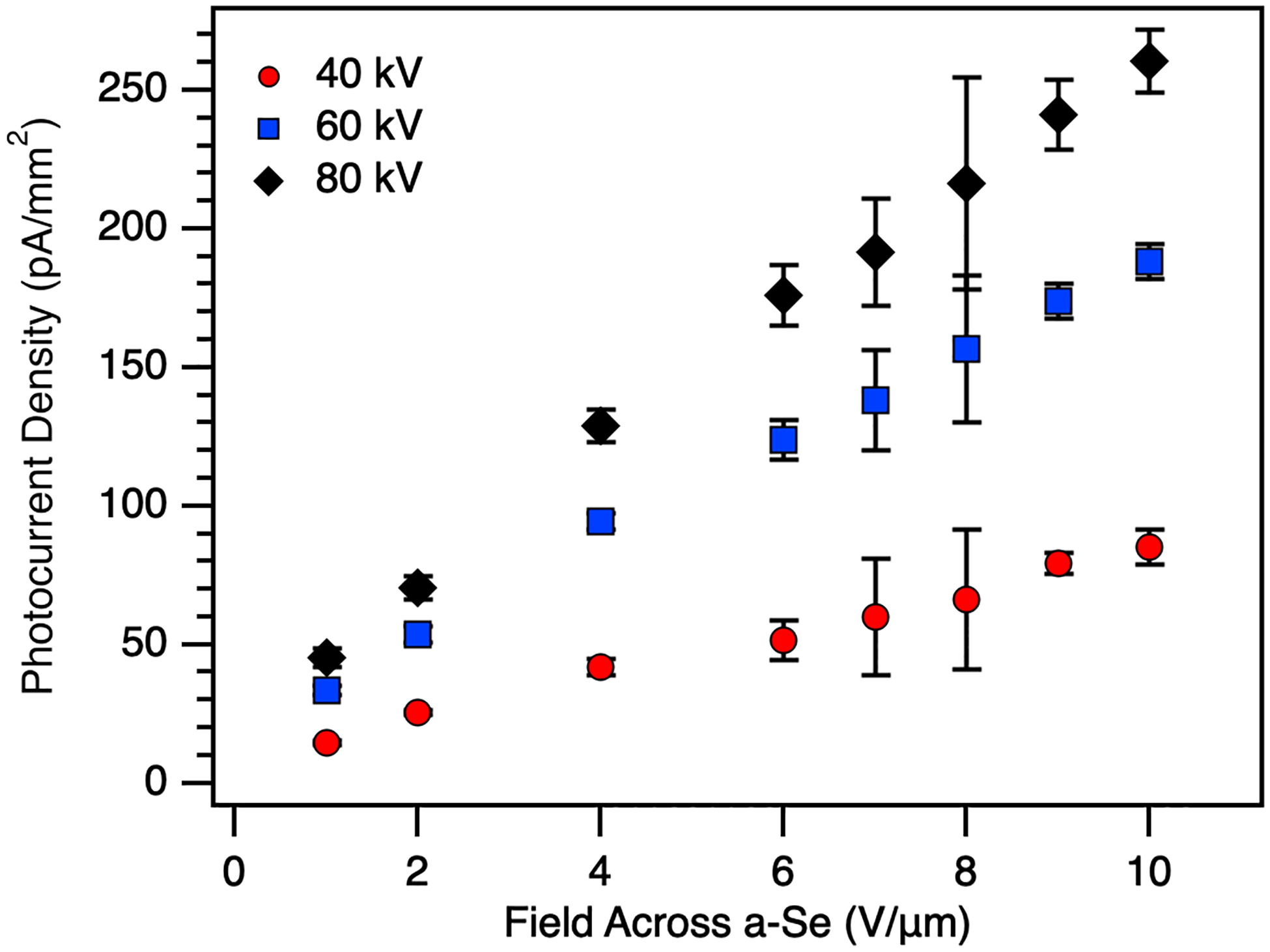

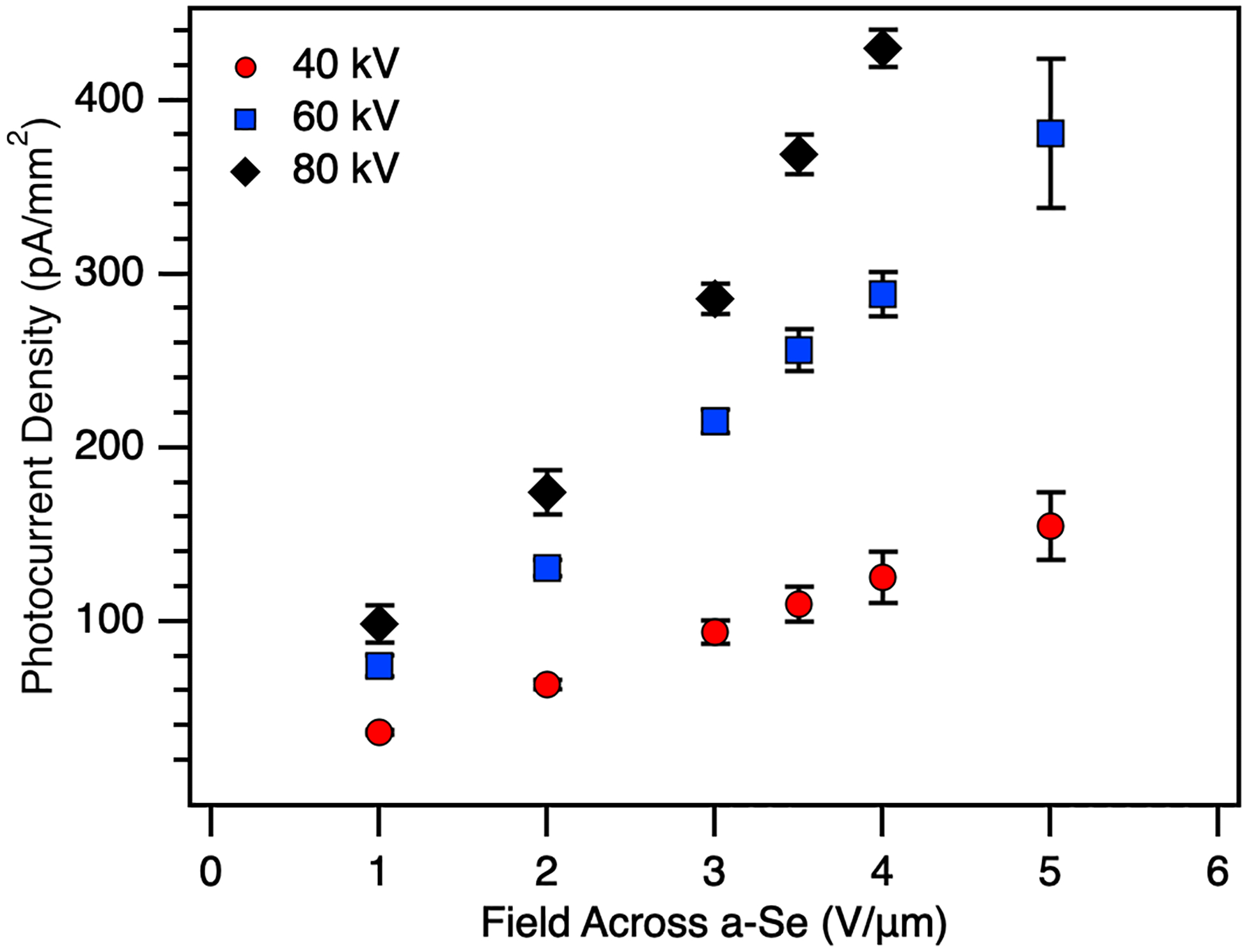
Photocurrent density as a function of applied electric field for a-Se devices a) 253 μm thick and b) 414 μm thick.

Fig. 7a and 7b show the X-ray photocurrent responses of the 253 and 414 μm devices, respectively. As the applied electric field increases, we see improved response from both devices. The 253 μm device demonstrates a linear increase in photocurrent as a function of field, while the 414 μm device shows a slightly more exponential trend as the applied field increases. This may be due to increased electron-hole pair (ehp) production in the thicker device, from greater absorption of higher energy photons. Higher electric fields will continue to improve the signal, as charge transport increases in a-Se increases and the probability of recombination is reduced. The increased photoresponse to increasing tube voltage in both devices comes from both the increase in total photon flux and the increase in ehp production with increasing photon energy.

**Fig. 8.**
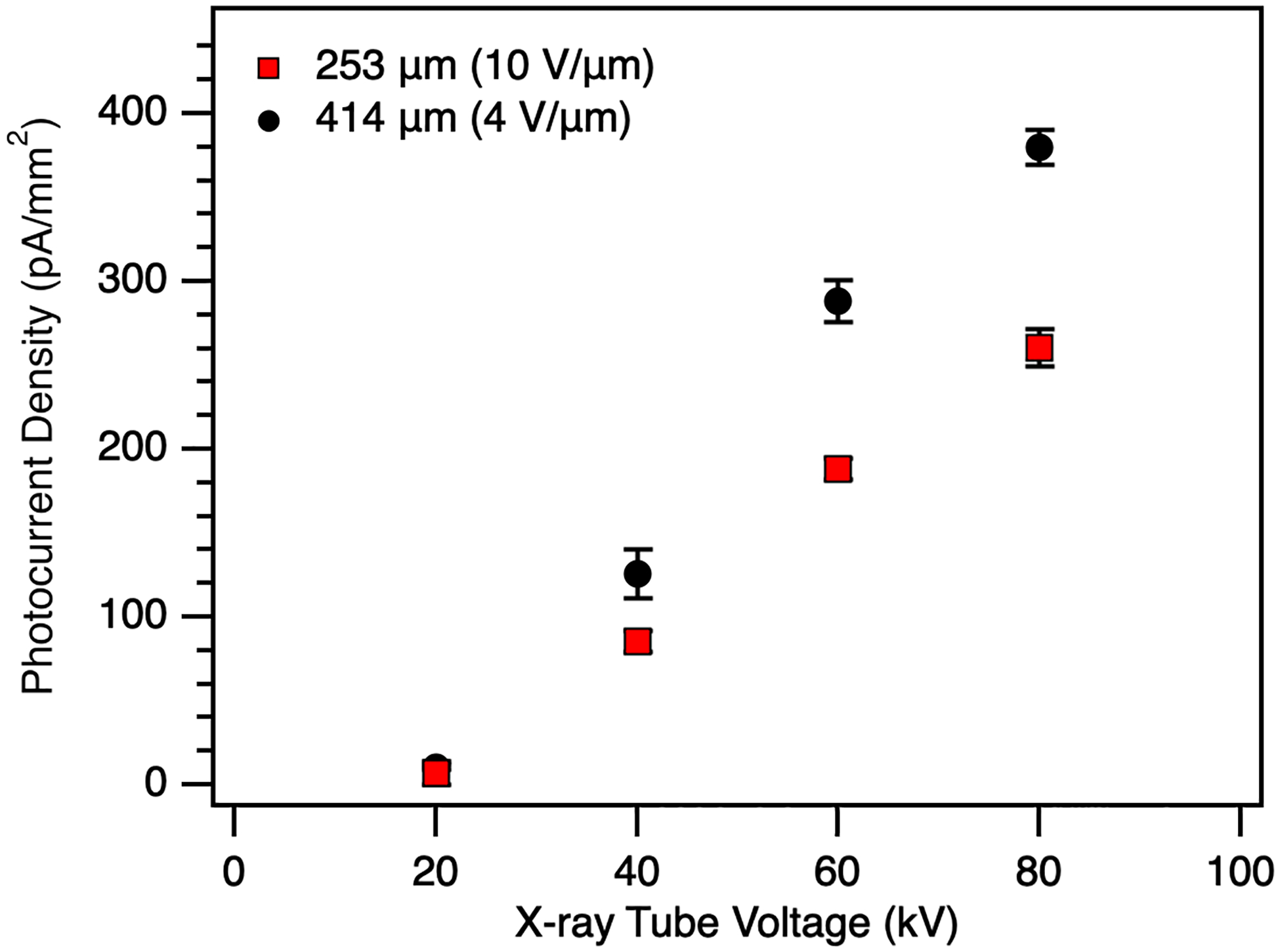
Photocurrent density of the 253 μm device biased at 10 V/μm and the 414 μm device biased at 4 V/μm as a function of the X-ray tube voltage.

Fig. 8 directly compares the photocurrent density as a function of tube voltage for each device at the maximum field for which data at each tube voltage was obtained. It can be seen that, even at a lower electric field, increasing the thickness of the a-Se increases the signal output with increasing tube voltage, as the higher energy X-rays are more greatly absorbed. Increasing the applied field to the 414 μm device will further increase signal output, far surpassing the performance of the 253 μm device. It should be noted that to achieve a field of 10 V/μm, a 400 μm layer requires an applied voltage of above 4 kV; the potential of the high voltage bias on the top surface of a-Se may damage the detector [37]. Additional capping layers and development of readout circuitry that can sustain these high voltages are required, and research into both is currently underway.

**Fig. 9.**
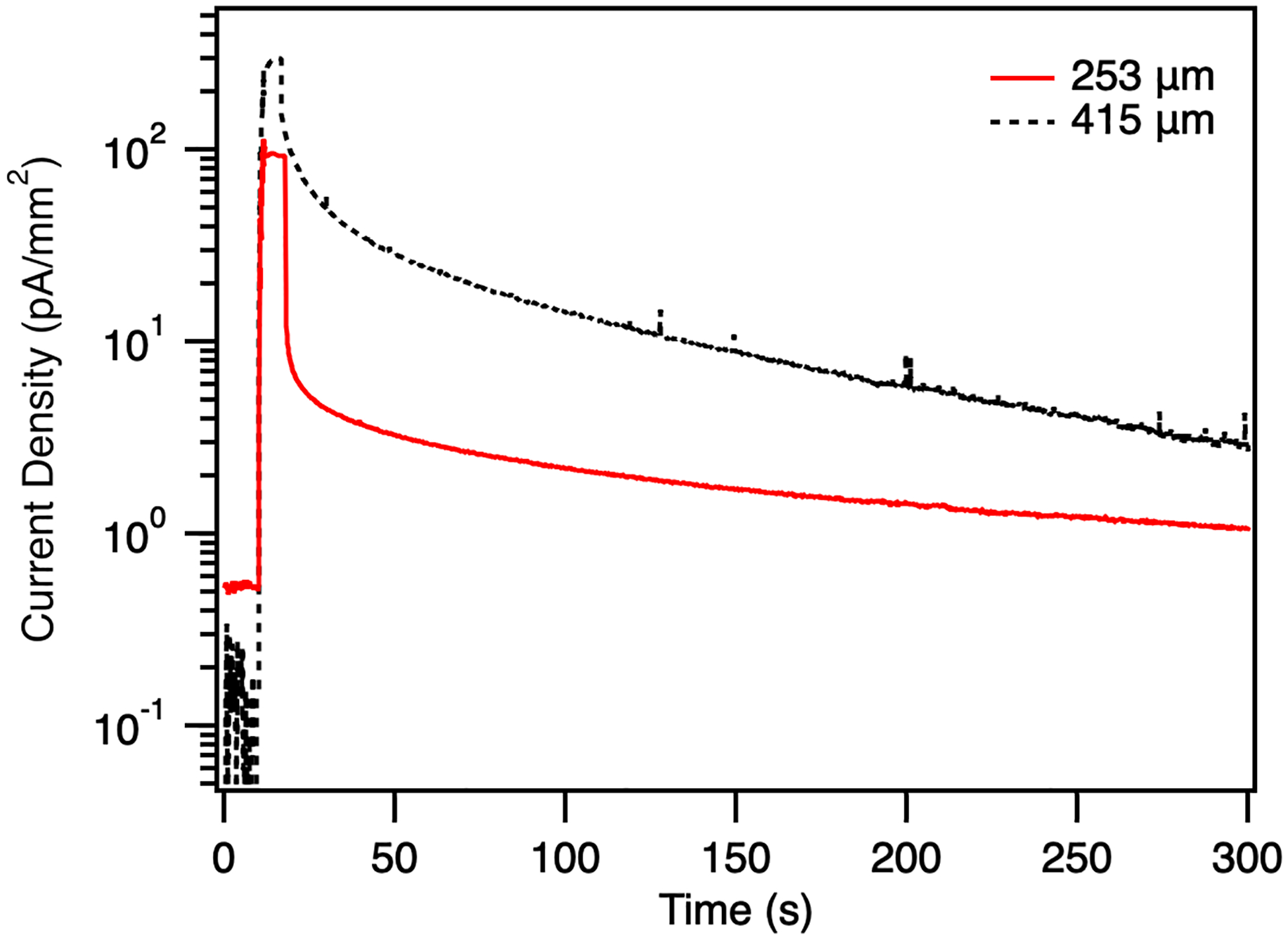
Comparison of the current density before, during, and after exposure to 60 kV tube voltage for the 253 μm and 414 μm devices biased at 4 V/μm.

A comparison of the signal and recovery for each device biased at 4 V/μm and exposed at 60 kV tube voltage is shown in Fig. 9. While the 414 μm device shows lower dark current and higher signal, the post-exposure recovery time is greatly extended compared to the 253 μm device. Both devices experience a long time to full recovery; approximately 15 minutes. The applied field of 4 V/μm is particularly low for a-Se, which is known to have reduced recovery times as the field increases and transport properties improve [38]. Any additional deep traps that are introduced with increasing thickness will further effect residual photocurrents, as carriers slowly recombine. Additionally, any reduction in X-ray fluence should improve temporal performance, as may be expected in practical applications. Even with optimization, the trade-off between temporal and signal performance must be considered for selecting the optimum thickness of the a-Se.

## IV. Conclusion

We have experimentally characterized thick a-Se photoconductors in the 250 μm and 400 μm range, targeting the low energy (LE), direct conversion layer requirements of a dual-layer X-ray detector. Mobility measurements across 100–414 μm thicknesses confirm that, although thicker layers exhibit reduced hole mobility at low fields (< 5 V/μm), mobility converges to previously reported values at fields above 5 V/μm. Dark current densities remained below the 10 pA/mm^2^ medical imaging threshold across all measured fields, enabled by the incorporation of a polyimide hole blocking layer. X-ray photoresponse measurements showed that increasing the a-Se thickness improves signal output at higher tube voltages, consistent with enhanced absorption of higher-energy photons. The 414 μm device demonstrated lower dark current and higher X-ray sensitivity than the 253 μm device under identical bias, although exhibited longer post-exposure recovery times. Future work will employ interrupted-field TOF experiments, delving deeper into how film quality is influenced as layer thickness increases, and if the additional interface from multiple depositions results in deep traps that reduce carrier transport.

These results validate that thick a-Se layers in the 200–400 μm range can deliver transport and noise performance that is equivalent to that of commercially available direct conversion detectors when operated above 5 V/μm. This prepares future work for the integration of thick a-Se layers into a large area TFT for a direct conversion, LE detector.
